# Clinical Audit on the Knowledge and Confidence of Medical Staff in Transgender Patient Management

**DOI:** 10.1192/bjo.2026.11757

**Published:** 2026-06-30

**Authors:** Prabin Gautam, Rachel Daly, Wan-Ting Yew

**Affiliations:** Kent and Medway Mental Health NHS Trust, Dartford, United Kingdom

## Abstract

**Aims:**

The primary aim of this project was to assess the current knowledge and confidence levels of medical employees regarding the management of transgender patients within the Kent and Medway Mental Health NHS Trust (KMMH). Against a background of recent legal changes and scarce guidelines, we hypothesised that despite likely clinical exposure to this demographic, staff confidence and awareness of specific policies would be low. The results are intended to drive the development of a dedicated Trust policy and training curriculum.

**Methods:**

This project was designed as a service evaluation utilising an anonymous electronic questionnaire distributed to medical staff by the medical education team. The survey consisted of five questions addressing clinical experience, self-reported confidence, training history, and awareness of gender reassignment processes and relevant guidelines. Qualitative data were also collected through free-text comments. Data collection and analysis focused on quantifying the gap between patient contact and provider preparedness.

**Results:**

A total of 43 completed responses were received from over 300 medical staff. The data revealed that while the vast majority of staff (83.7%) had been involved in the care of transgender patients, confidence remained low. Only 41.9% of respondents felt “quite confident” managing these patients in a mental health setting, with 58.1% reporting they were “not confident”.

Gaps in education and governance were prominent. Eighty-six per cent (86%) of respondents stated they had received no training in the management of transgender patients. Furthermore, 60.5% were unaware of current gender reassignment processes in the United Kingdom (UK), and 79.1% were unaware of any national guidelines or hospital policies regarding healthcare for this group. Qualitative feedback supported these figures, with staff requesting specific training on pronouns, legal frameworks, and “user perspectives” from the trans community.

**Conclusion:**

The audit demonstrates a significant disparity between the high frequency of clinical contact with transgender patients and the low levels of staff training and confidence. The widespread lack of awareness regarding existing guidelines indicates an urgent need for the formalisation of Trust policies and the implementation of targeted educational workshops. Improving staff knowledge is critical to mitigating risk and ensuring effective, respectful care for transgender service users.

